# Feasibility of chest ultrasound up to 42 m underwater

**DOI:** 10.1186/s13089-023-00334-5

**Published:** 2023-08-21

**Authors:** Matteo Paganini, Giuseppe Cantarella, Danilo Cialoni, Ezio Giuffrè, Gerardo Bosco

**Affiliations:** 1 Department of Biomedical Sciences, University of Padova, Via Marzolo, 3, 35131 Padua, Italy; 2 Department of Physics, Informatics and Mathematics, University of Modena and Reggio Emilia, Modena, Italy; 3 H-2 Tech, Padua, Italy

**Keywords:** Underwater medicine, Diving medicine, Extreme environments, Physiology, Environmental physiology, Ultrasound, Lung ultrasound, Chest ultrasound

## Abstract

After recent advancements, ultrasound has extended its applications from bedside clinical practice to wilderness medicine. Performing ultrasound scans in extreme environments can allow direct visualization of unique pathophysiological adaptations but can be technically challenging. This paper summarizes how a portable ultrasound apparatus was marinized to let scientific divers and sonographers perform ultrasound scans of the lungs underwater up to − 42 m. A metallic case protected the ultrasound apparatus inside; a frontal transparent panel with a glove allowed visualization and operation of the ultrasound by the diving sonographer. The inner pressure was equalized with environmental pressure through a compressed air tank connected with circuits similar to those used in SCUBA diving. Finally, the ultrasound probe exited the metallic case through a sealed aperture. No technical issues were reported after the first testing step and the real experiments.

## Background

Ultrasound is a non-irradiating technique widely accepted as a standard of care in almost every healthcare sector. As a diagnostic tool, ultrasound was first developed to explore anatomic areas in which standard radiography had limited applications and accuracy, such as the abdomen, the heart, arteries, and veins. Surprisingly, ultrasound has become a fundamental tool for clinicians. After creating portable apparatuses, point-of-care ultrasound (POCUS) has implemented the traditional physical exam so that real-time insonation and interpretation allow the clinician to confirm or reject diagnostic hypotheses at the bedside [1].

Practical applications of POCUS encompass most medical specialties. For example, after being integrated into radiology and cardiology, POCUS registered a steep increase in emergency medicine and intensive care due to the ability to rapidly answer simple but fundamental questions in the critically ill [2]. Also, thanks to device dimensions, battery duration, and harsh environments endurance improvements, POCUS has been progressively introduced in disaster medicine, military medicine, and wilderness medicine [3].

Such peculiar features of POCUS allowed its introduction in the study of pathophysiological adaptations of humans to extreme environments. For example, in the last decade, high-altitude physiology has been increasingly investigated with portable ultrasound devices [4], showing promising results for the early detection of high-altitude pulmonary edema [5] and conflicting evidence regarding the diagnosis of high-altitude cerebral edema through optic nerve sheath diameter measurement [6].

Similarly, ultrasound has been used in diving medicine initially to detect acute variations, mainly in the cardiovascular system. One consequence is that the immersed human body develops a series of autonomic responses entailing, for example, blood shift from the peripheral circulation—pooling into the chest and preserving brain perfusion while diving—bradycardia, and splenic contraction. Altogether, these responses to immersion and submersion are referred to as *mammalian reflex* [7] since it is shared among different mammals. Also, divers are prone to develop decompression sickness (DS), especially when decompression from depth while diving using breathing apparatuses does not follow safety measures or when subjects have strong predisposing factors to DS development [7]. The causative mechanism entails bubble formation in the microvascular system, paired with evidence demonstrating the potential role of activated platelets [8]. In light of the constantly growing number of subjects practicing diving with different techniques, hyperbaric and diving medicine knew tremendous development in recent years in the attempt to understand better, prevent, and treat the derangements mentioned above.

With this perspective, POCUS has been used to detect venous gas emboli traveling from peripheral circulation to the heart when resurfacing after self-contained underwater breathing apparatus (SCUBA) dives [9, 10] and signs of pulmonary edema after breath-hold diving (BHD) [11–13].

The pursuit of investigating unique cardiovascular adaptations experienced by humans in the underwater environment, such as the mammalian reflex, pushed physiologists to marinize ultrasound devices and allow the visualization of the heart underwater through echocardiography [14–17]. However, such experiences have been conducted only up to 10 m underwater.

In recent experiments, Bosco et al. investigated oxygen levels derangements in the arterial blood of breath-hold divers through blood gas analysis performed at the surface, at 15 and 42 m underwater, and after resurfacing; pulmonary atelectasis or pulmonary interstitial edema was suggested as the causing factor [18, 19]. The same research group integrated arterial blood gas analysis and lung ultrasound to verify such hypotheses but had to overcome the challenge of marinizing an ultrasound apparatus to reach 42 m underwater. Here we describe the design, test, and use of an insulating device to protect the ultrasound apparatus underwater and the experiments carried out.

## Materials and methods

The insulating device was similar to another one patented and used in previous experiments [20], but with different shapes. It was tested and used up to − 42 m of freshwater (mfw).

**Fig. 1:**
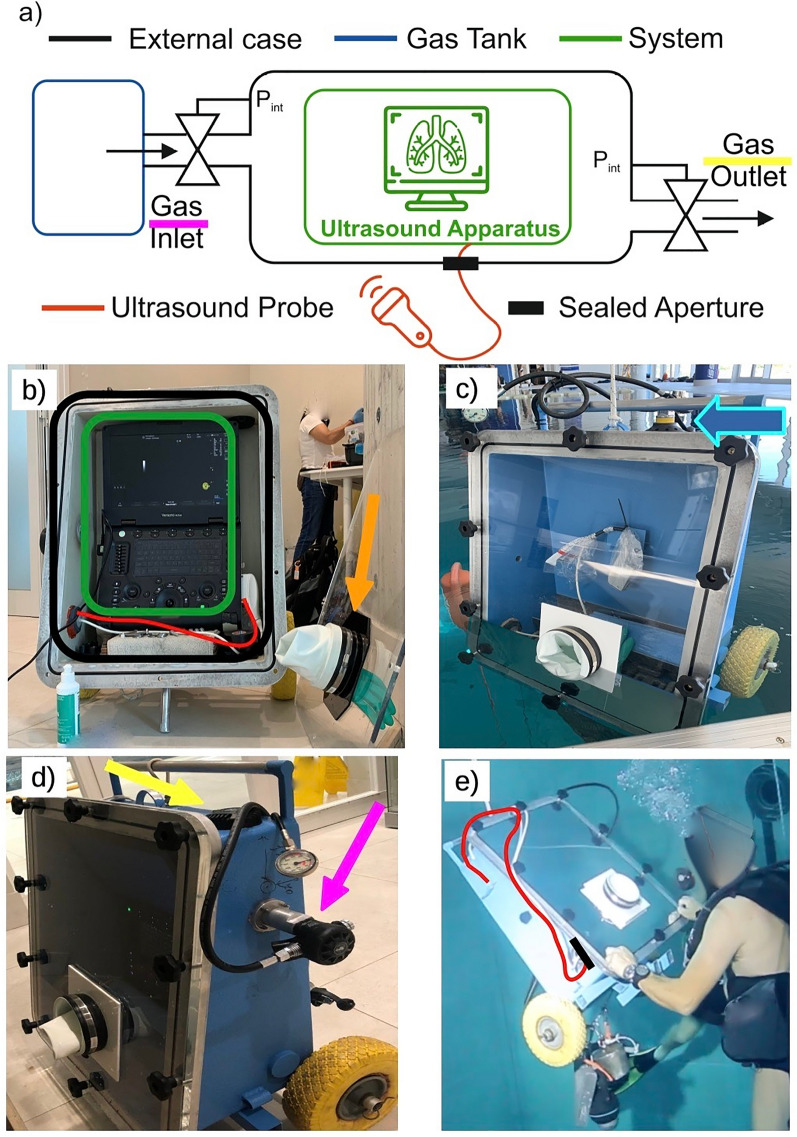
The insulating device used to allow underwater chest ultrasound. **a** Schematic representation of the setup. **b** The opened case (black), with the ultrasound apparatus inside (still charging; green) and the ultrasound probe connected (red); the orange arrow indicates the frontal panel and the glove used to operate the ultrasound. **c** First test of the empty case; the blue arrow indicates the air tank. **d** Sides of the insulating device with the wheels used to move it, the inlet circuit (purple arrow), and the outlet valve (yellow arrow). **e** One of the authors (EG) testing the device underwater; on the right side, the ultrasound probe (red line) sticks out of the metallic case through a dedicated sealed aperture (black line)

The concept required the creation of a waterproof box resistant to varying environmental pressures and temperatures and protecting the ultrasound apparatus from accidental impacts with objects underwater. To properly visualize images, one side of the box had to be transparent, keeping sufficient resistance to the hazards mentioned above. Furthermore, the need to access the keyboard—to adjust the ultrasound settings, start video recordings, and create new folders for each patient—required a sufficiently resistant but handy device in the frontal panel. For a schematic representation of the whole device, please see Fig. 1a.

In detail, the setup was composed of a metallic external case (width × height × depth 60 × 75 × 30 cm, wall thickness of 6 mm, weight 35 daN) made of Anticorodal 6060 (Fig. 1b: black rectangle), with two rubber wheels at the bottom to facilitate movements and without a frontal panel. The frontal borders of the metallic case contained a rubber gasket to improve insulation. The weight of this setup was 35 daN and had a buoyancy of 40 daN. The case contained a removable, portable ultrasound apparatus (Versana Active, GE Healthcare, United States; Fig. 1b: green rectangle) equipped with a sector probe (1–4 MHz) of the same manufacturer (Fig. 1b, e: red line), which passed through a sealed aperture in the case, thus allowing real-time scans of the subjects. To ensure proper machine–user interaction, a transparent panel made of polycarbonate (width × height 60 × 75 cm, wall thickness of 3 cm; Fig. 1b: orange arrow) was mounted on the front side of the metallic case. In addition, a right-handed rubber glove (thickness: 2 mm) was applied on a circular opening in the frontal panel to allow interaction with the keyboard. Every time the ultrasound apparatus was marinized—after fully charging the battery and connecting the probe—the panel was applied to the case and sealed by fastening 10 threaded devices.

An important concept to bear in mind is that environmental pressure underwater has a constant increase of about 1 absolute atmosphere (ATA) every 10 m of water, plus 1 ATA already existent at the surface and due to the atmosphere weight (assuming experiments at sea level) [7]. From that, the need to ensure internal equalization with environmental pressure to avoid water infiltrations and disconnections or tearing of the rubber glove. The case was therefore connected to a standard diving cylinder with 200 atm of compressed air using a circuit similar to that used in SCUBA diving. Specifically, a first stage was attached to the scuba tank valve and reduced tank air pressure to intermediate values, routing it to a regulator hose (Fig. 1c: blue arrow). A second stage valve (commonly known in diving as “mouthpiece”) connected the regulator hose and the case inlet (Fig. 1d: purple arrow). It kept the inner pressure stable by forcing air inside the watertight case during the descent at the same pressure as the surrounding water environment.

Conversely, during ascent, the inner pressure becomes higher than the external, with the same reduction of 1 ATA every 10 m of water. To avoid the explosion of the rubber glove during ascent, a commercially available gas outlet valve (of the type used in drysuits to avoid overpressurization) was placed directly on the case and allowed a controlled air leak, ensuring a safe pressure equalization until resurfacing (Fig. 1d: yellow arrow).

### The experiments

The experiments occurred at the world’s deepest pool, “Y-40 THE DEEP JOY”, with a water temperature of 31.5 ± 0.5 °C in Montegrotto Terme (Padova, Italy).

Preliminary pilot testing sessions were carried out in April–May 2021, submerging the whole apparatus without the ultrasound device to evaluate the water-tightness and prevent potential other issues. After some water drops were detected inside the frontal panel, the insulation was improved by greasing the gasket before the frontal panel application. However, a “near miss” accident happened when the apparatus was manually lowered, and the equalization failed: the glove dangerously inflated but did not break. The cause was attributed to the too-fast lowering of the device. To avoid similar events, the insulating device was slowly lowered into the water using a winch, with a SCUBA diver constantly monitoring for proper equalization and ready to stop the maneuver (Fig. 1e).

The ultrasound apparatus was inserted into the case in the second feasibility step. The risk of overheating and malfunctioning of the ultrasound apparatus resulted from the environmental temperature (warm thermal water), the increase of inner pressure while descending (known to increase temperature), and the ultrasound apparatus itself. No issues were reported during the activities, and the ultrasound properly worked during all the sessions.

**Fig. 2:**
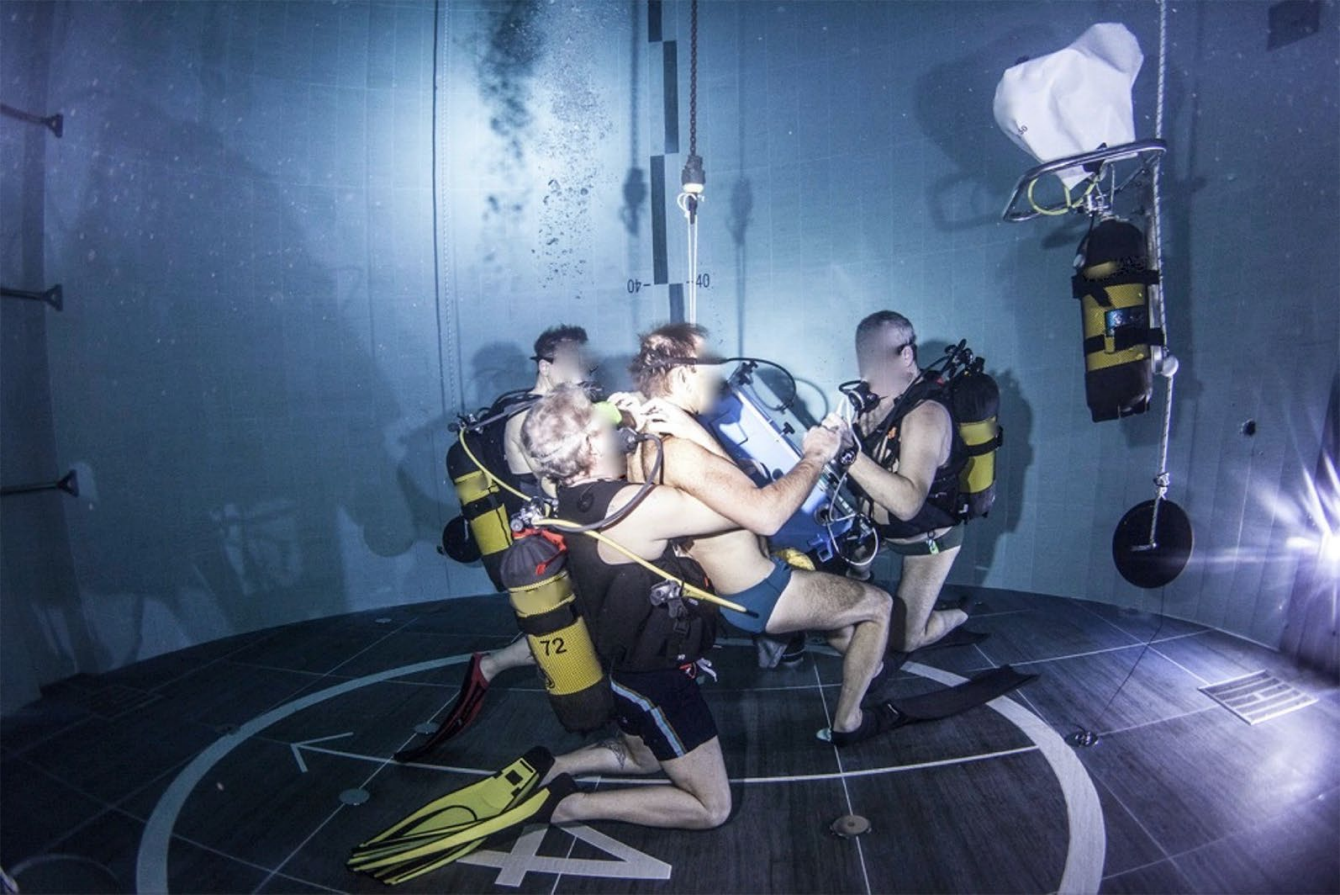
Chest ultrasound performed by the authors at − 42 m underwater

Experiments were carried out at − 15 mfw and − 42 mfw; therefore, operational pressures (internal and external) of 2.5 and 5.2 ATA were estimated during the projecting phase and guided the choice of materials and their dimensions and thickness. The marinized ultrasound apparatus was submersed at each depth before every experimental session using a winch and accompanied by a SCUBA diver to monitor for any leakages or technical failures. Once at depth, each lung scan lasted between 40 and 50 s (Fig. 2). Total operation time was about one hour, including the descent, bottom time, and resurfacing. At the end of each experimental session, the apparatus was winched out of the water, with a SCUBA diver again monitoring the proper functioning of the gas outlet valve and pressure equalization.

## Conclusions

The recent diffusion of diving sports in the population asked for a better comprehension of pathophysiological changes occurring in humans while underwater. As previously noted in other preclinical and clinical settings, POCUS proved useful in investigating physiology and pathology in environmental medicine, specifically in diving and underwater medicine. Furthermore, the watertight case described in the present paper safely protected the ultrasound apparatus during underwater experiments up to − 42 mfw.

Further experiments could increase the understanding of human adaptations to the underwater environment to avoid and mitigate the hazardous consequences of hypoxia during breath-hold diving and explore strategies to prevent acute diseases in SCUBA and technical divers.

## Data Availability

Not applicable.

## Abbreviations

POCUS: Point-of-care ultrasound
DS: Decompression sickness
mfw: Meters of freshwater
SCUBA: Self-contained underwater breathing apparatus
BHD: Breath-hold diving
ATA: Absolute atmosphere
